# The Effect of the Fear of COVID-19 on Healthcare Professionals’ Psychological Adjustment Skills: Mediating Role of Experiential Avoidance and Psychological Resilience

**DOI:** 10.3389/fpsyg.2020.561536

**Published:** 2020-10-21

**Authors:** İsmail Seçer, Sümeyye Ulaş, Zeynep Karaman-Özlü

**Affiliations:** ^1^Faculty of Education, Counseling and Guidance, Atatürk University, Erzurum, Turkey; ^2^School of Health, Gümüşhane University, Gümüşhane, Turkey; ^3^Faculty of Nursing, Atatürk University, Erzurum, Turkey

**Keywords:** fear of COVID-19, psychological adjustment, experiential avoidance, psychological resilience, healthcare professionals

## Abstract

As the COVID-19 outbreak is rapidly spreading all over the world, it’s secondary consequences will negatively affect both societies and individuals. The target group, expected to be exposed to the secondary negative consequences most intensely during the pandemic process and afterward, is undoubtedly the healthcare professionals. In this research, the impact of the fear that healthcare professionals in Turkey developed against the outbreak of COVID-19 on their psychological adjustment skills is examined, and in this context, the mediating role of experiential avoidance and psychological resilience is examined. In this context, an answer was sought for the question “Does experiential avoidance and psychological resilience have a mediating role in the impact of COVID-19 fear on psychological adjustment skills of healthcare professionals?” The research was carried out with a total of 370 healthcare professionals reached via online data collection method. Structural equation modeling was used in the data analysis process, and as a result, it was determined that the fear of COVID-19 had a negative effect on the psychological adjustment in healthcare professionals; however, psychological resilience was found to have a protective function that limits this effect, and experiential avoidance has a risk factor that aggravates this effect. Findings obtained from the research are discussed in the context of the literature.

## Introduction

The COVID-19 epidemic started in late 2019 in China, spread rapidly throughout the world, and has affected both societies and individuals in many aspects. After being described as a pandemic by World Health Organization [WHO] (2020), a wide variety of prevention and treatment approaches have been applied worldwide. Applying precautions such as social distancing and strict quarantine in many countries especially in China, Italy, Spain, and Turkey has become one of the most basic tools used to limit the spread of the disease.

Despite all kinds of precautions, millions of people worldwide have been infected with this disease (World Health Organization [WHO], 2020). However, the number of those who recovered have been one and a half million (World Health Organization [WHO], 2020). The number of people who died due to the pandemic has been more than 200,000. The burden of all individuals infected, treated, and returned to their normal life or passed away is on the shoulders of healthcare professionals all over the world. Healthcare professionals have to identify the people infected with the disease, respond to their treatment needs, carry out the severe and difficult treatment processes in hospitalized patients, face the psychological breakdown created by each patient passed away and also face the risk of developing the disease at any time. Each mentioned situation is a difficult living condition in itself, and these conditions are expected to create secondary consequences for healthcare professionals in the short- and long-term. Banerjee (2020) and Ornell et al. (2020) stated that there is an important possibility to see the secondary consequences in every aspect of the society during pandemic periods and that emotional and behavioral problems such as *anxiety, fear, depression, suicide, substance abuse*, etc. may come to the fore among them. In this context, it is thought that the healthcare professional, who are at the forefront of the fight against the pandemic, have an unwanted but important possibility to develop the secondary symptoms in addition to the possibility of getting infected with the virus.

Individuals’ responses to challenging living conditions can generally be as shock, panic, acute stress, post-traumatic stress disorder, grief disorder, anxiety disorder and depression, etc. (Aydın, 2020). Each of these forms of response directly points to the individual’s psychological adjustment skills. If psychological adjustment is considered as the ability of the individual to cope with daily life difficulties, to control intense anxiety, depressive symptoms, and stress factors, it can be said that traumatic and challenging living conditions can have an effect that forces the psychological adjustment skills of the individual. In this context, it can be interpreted that the difficult life conditions experienced by healthcare professionals due to the COVID-19 outbreak may put them at a disadvantage and trigger various psycho-social problems in the context of psychological adjustment skills.

In this context, it can be said that the first negativity expected to threaten the psychological adjustment skills of the healthcare professionals is the fear developing due to COVID-19. Fear is a defense mechanism of an individual against dangerous situations and includes the basic responses of the individual in order to survive and protect themselves against these threatening situations. However, the disproportionate level of fear can predispose to various psychopathologies (Shin and Liberzon, 2010; Garcia, 2017; Shigemura et al., 2020; Wang et al., 2020). Even in healthy individuals, there may be a risk of densification of symptoms such as stress, and thus establishing an environment for psychological disorders (Ornell et al., 2020; Shigemura et al., 2020). Although there is no definite epidemiological data regarding the psychological effects of COVID-19 on individuals and its effect on public health, the results of the limited studies show that the fear of getting COVID-19 leads to intense emotional and behavioral consequences such as boredom, loneliness, anxiety, sleep problems and anger (Brooks et al., 2020b). The results of studies indicate depression, anxiety disorders, post traumatic stress disorder (PTSD), paranoid and psychotic disorders, and even suicide among the emotional behavioral consequences of this fear (Xiang et al., 2020). Considering the fact that healthcare professional may also be susceptible to various psychopathological conditions, it may be thought that the potential risk situation will increase even more. Hence, the data related to the literature indicate that traumatic and challenging living conditions can be more common in individuals with prior psychological disorders (Wang et al., 2020; Park and Park, 2020). Research results on the former Ebola-like outbreaks also support this view (Reardon, 2015; Shigemura et al., 2020). Even if the pandemic periods are over, secondary psycho-social effects expected to occur in healthcare professional who experience trauma closely, and it may affect the individual’s quality of life for a long time (Shultz et al., 2016). Therefore, it can be expected that the fear that healthcare professionals develop in this process will have a negative effect on their psychological adjustment skills by triggering various psychopathological symptoms.

There are also some characteristics that strengthen or make the individual’s position disadvantageous in the face of difficult living conditions. In this context, experiential avoidance can be shown as an important determining variable among the variables that shape the level of exposure of the individual to challenging life events. Experiential avoidance is defined as reluctance to experience emotions, thoughts, moments and physical feelings that are considered negative and avoidance responses to reduce the frequency or effect of these experiences (Hayes et al., 1996). It is also expressed as the rigid and unchangeable attitude that the individual adopts in the face of negativities and is associated with various psychological problems in this aspect (Ottenbreit and Dobson, 2004). This concept, which includes both different experiences avoided and different strategies used for avoidance, also covers the cognitive, emotional and behavioral dimensions of avoidance. In this sense, it is thought that experiential avoidance has important effects on the psychological adjustment skills of the individual in the short- and long-term. That is, facing negative situations, the individual often uses a number of ways such as paying attention to another direction, denial and repression, but these ways can prepare an environment for the effects of the negativity avoided in the long run to continue and the problems associated with it to become widespread (Briggs and Price, 2009; Hayes et al., 2012). Accordingly, it can be said that the possible avoidance responses due to the fear of COVID-19 can play an important role in the emergence and persistence of many psychological problems. There are only a limited number of studies addressing the psychological effects of the COVID-19 outbreak on individual and public health, as the problem is still new. However, limited studies indicate that individuals show severe signs of adjustment disorders (Ornell et al., 2020; Shigemura et al., 2020). Individuals naturally will try to get rid of this problem through effective coping strategies. However, the secondary effects developing due to the pandemic may become chronic in individuals who show avoidance reactions with the effect of various psycho-social factors. The data related to the literature support this idea. For example, Santanello and Gardner (2007) and Mahaffey et al. (2013) determined that individuals with high experiential avoidance have intense anxiety disorders. Cribb et al. (2006) and Briggs and Price (2009) determined that they have depression. Rawal et al. (2010) determined that they have eating disorders, Orcutt et al. (2005) determined post-traumatic stress disorders, and Machell et al. (2015) determined that low level of subjective well-being. Therefore, it can be argued that the healthcare professionals’ avoidance responses, which we can define as the dysfunctional coping approaches, are a risk factor that can disrupt psychological adjustment skills in the short- and long-term.

Despite the risk factor expected to be experienced in healthcare professionals through the experiential avoidance, psychological resilience can be demonstrated as a feature that strengthens the positions of the healthcare professionals against the adverse effects caused by the COVID-19 outbreak, and it enables them to cope effectively both personally and professionally. Psychological resilience has been defined by Brooks et al. (2020a) and Earvolino-Ramirez (2007) as the ability of the individual to quickly rally, recover and return to pre-crisis status after being hurt. Similarly, it is defined as the ability of the individuals to be able to return to the status that enables them to be successful in uncertain and challenging processes (Luthans et al., 2006; Seçer and Ulaş, 2020a) and to fulfill the tasks and behaviors expected from them (Öz and Yılmaz, 2009). From this point of view, psychological resilience can be seen as an important protective function in professions serving in traumatic processes including healthcare professionals (Brooks et al., 2020a), and in this respect, it can be thought that it has an effect that prevents the psychopathologies developed due to the COVID-19 process from becoming chronic and limits its dimension of threatening the life of the individual in a short- and long-term.

In line with the information related to the literature given above, it is clear that the fear of COVID-19 poses a significant risk for its potential to disrupt healthcare professionals’ psychological adjustment skills. This risk can be expected to deepen in healthcare professionals with experiential avoidance. On the other hand, it is thought that psychological resilience can strengthen the position of healthcare professional in dealing with the negativity caused by the epidemic. Accordingly, in this research, the effect of fear of getting COVID-19 on the psychological adjustment levels of healthcare professionals was examined through the mediating role of experiential avoidance and psychological resilience. The results of the research are expected to contribute to the understanding of the nature and consequences of secondary health problems likely to develop due to the COVID-19 in healthcare professionals as well as to expand our perspective on understanding individual risks and protective factors. It is possible that this broadening in our perspective will have important consequences for the development and implementation of preventive and rehabilitative practices for healthcare professionals after the pandemic. In this direction, answers to the questions given below were sought within the scope of the research.

(1) What is the general view of psychological adjustment skills in healthcare professionals?
(2) Does the fear of COVID-19 have a direct predictive effect on psychological adjustment in healthcare professionals?
(3) How is the effect of COVID-19 fear on psychological adjustment shaped in healthcare professionals after the variables of experiential avoidance and psychological resilience were added to the model?

## Materials and Methods

### Participants

Participants of the research consist of 390 healthcare professionals aged between 20 and 65 years (*m* = 16.40, SD = 2.14). 73.3% of the participants are females, 25.2% are males, and 1.5% are those who did not indicate their genders. In reaching the participants, an online data collection process was used. In this context, the data were collected from a total of 390 healthcare professionals (doctors, nurses, pharmacists, health officers, medical attendants, etc.) by reaching them from the healthcare organizations in different regions of Turkey through a convenient sampling method. In this context, especially the relevant hospital administrations were contacted and they were asked to direct the online data collection link to the personnel they deem appropriate. Forty-five percent of the healthcare professionals constituting the participants are married, 52.4% are single, and 7% are in the divorced-separate category. In addition, 14.7% of the participants have at least one chronic condition (In the personal ınformation form, it was asked “Have you have a psychological or medical illness?” and data on 17 healthcare professionals who stated that they had a psychological illness were not included in the analysis) and 58.7% of them have at least one task related to COVID-19 in the hospitals they work. Considering their assigned positions, 49.3% of the participants work in other services other than intensive care and outpatient clinics (Dialysis Unit, Chemotherapy Unit, Blood Center, etc.), 20.2% in emergency services, 16% in intensive care services, 8.8% in outpatient services, and 5% in ambulance services.

### Measures

#### The Fear of COVID-19 Scale

The Fear of COVID-19 Scale is a self-report based assessment tool consisting of seven items and one dimension developed by Ahorsu et al. (2020) to assess the anxiety and depressive symptoms that develop due to the COVID-19 outbreak in individuals. The scale is a four-point Likert type (never, rarely, often, and always) for individuals in the age group 18 and over (Sample questions are like “I am very afraid of coronovirus and talking about coronovirus bothers me”). The scale was adapted to Turkish culture for adults by Satici et al. (2020). The scale preserved the seven items in its original form in Turkish culture (χ^2^/SD = 2.10, REMSEA = 0.041, RMR = 0.037, SRMR = 0.040, CFI = 0.99). The internal consistency value of the scale was calculated as Cronbach Alpha 0.91. The scores that can be obtained from the scale range from 7 to 28. The high scores indicate the high level of fear of coronavirus.

#### Experiential Avoidance Scale

Experiential Avoidance Scale is a self-reporting four-point likert type (never, rarely, often, and always) assessment tool adapted to Turkish culture (Ekşi et al., 2018) and developed to determine the avoidance responses of individuals against various experiences (Sahdra et al., 2016). The sub-dimensions included in the scale are *behavioral avoidance, distress aversion, procrastination, distraction/suppression, repression/denial*, and *distress endurance* (Sample questions are: “Even if it is very little, I avoid activities that may hurt me and avoid situations where I may feel nervous”). There are five items in each sub-dimension and the scale consists of 30 items in total. The scores that can be obtained from the scale range from 30 to 120. In the scale, only the scores of the sub-dimensions are calculated instead of the total score and the high scores indicate the problematic avoidance in the relevant sub-dimension. Within the scope of this research, the factor structure of the scale was reviewed based on the data obtained from the study group and model fit indexes (χ^2^/SD = 2.41; REMSEA = 0.071, RMR = 0.073, SRMR = 0.070, CFI = 0.98) and internal consistency coefficient Cronbach alpha = 0.85 were determined to be sufficient.

#### Brief Resilience Scale

Brief Resilience Scale is a four-point likert type (never, rarely, often, and always) assessment tool developed by Smith et al. (2008) and adapted to Turkish culture by Doğan (2015). The scale consists of six items, and the high scores indicate a high level of psychological resilience. The scores that can be obtained from the scale range from 6 to 24 (Sample items are: “It does not take me a long time to come to myself after stressful situations and I will survive difficult times with very little trouble”). In this research, the construct validity of the scale was reviewed, and it was determined that the model fit indices (χ^2^/SD = 1.96; REMSEA = 0.062, RMR = 0.063, SRMR = 0.067, CFI = 0.98) were at a good level and internal consistency coefficient Cronbach alpha = 0.91 were determined to be sufficient.

#### Depression Anxiety Stress Scales

Depression Anxiety Stress Scales is a four-point likert type (never, rarely, often, and always) assessment tool developed by Lovibond and Lovibond (1995) to assess symptoms of depression, anxiety, and stress, and then revised to 21 items by Brown et al. (1997). The scale was adapted to Turkish by Yılmaz et al. (2017). The data on the construct validity of the scale (χ^2^/SD = 2.84; REMSEA = 0.051, RMR = 0.036, CFI = 0.98) showed that the three-factor structure with 21 items had a good fit level and internal consistency coefficient Cronbach alpha = 0.79 were determined to be sufficient (Sample questions are: “I felt scared even though there was no valid reason, and I was worried as I would panic and have egg on my face.”). The scores that can be obtained from the scale range from 21 to 84, and high scores indicate the high levels of the symptoms of depression, anxiety, and stress.

### Procedure and Data Analyses

The research initiated with obtaining permission to conduct the research from Gümüşhane University Health Sciences Ethics Committee, then, the necessary permissions were provided from the local administrators. In the data collection process, online tools were used due to the intensive working hours of the healthcare professional and social distancing restrictions. In this context, the online data collection link^1^ prepared via Google Forms was delivered to healthcare professionals through email and instant messaging apps. In this sense, healthcare professionals were contacted through the relevant hospital chief physicians and other relevant units, and additional explanations about volunteering and data confidentiality were also added to the online data collection link. Information regarding the fact that they can cancel filling the questionnaire at any time was also added. The online data collection process was completed within 15 days. Data collection and compilation were carried out by three researchers experts in health sciences, psychology, and psychological counseling. Since the data collection process was online, there was no data loss. On the other hand, when the parametric conditions were examined, it was determined that the data of 17 participants included extreme values that would disturb normality, and it was decided to exclude them from the data set.

In order to find answers to the research questions, structural equality analyses were carried out with the LISREL 9.2 software. In this context, the confirmatory measurement model was tested to examine the fit of the model constructed in the preliminary analysis. In the measurement model, one implicit variable was defined for the fear of COVID-19, experiential avoidance, psychological resilience, and psychological adjustment variables, and a total of 22 indicative variables were defined. The fit indices for the measurement model (χ^2^/SD = 1.60; REMSEA = 0.071, RMR = 0.073, SRMR = 0.073, NFI = 0.95, CFI = 0.97, GFI = 0.92) show that the constructed model was confirmed and that all implicit variables have a good level of agreement with the indicator variables they represent and other implicit variables (Tabachnick and Fidell, 2013; Seçer, 2015). At the stage after the verification of the measurement model, three different models created in the context of research questions were tested with the structural equation model. CFI, NFI, GFI, RMR, SRMR, RMSEA, and χ^2^ values, which are the fit indices frequently used in the structural equation model, were examined. In the evaluation of the model fit indices, different criteria were taken into account as suggested. In this context, Schumacker and Lomax (2004) and Seçer (2015) suggest that in the structural equation model, model fit indices should be ≥0.90 for acceptable fit and ≥0.95 for perfect fit for RFI, TLI, CFI, NFI, NNFI, and IFI. They suggest that model fit indices should be ≥0.85 for acceptable fit and ≥0.90 for perfect fit for GFI and AGFI, and ≤0.08 for acceptable fit and ≤0.50 for perfect fit for RMR, REMSEA, and SRMR.

## Results

**FIGURE 1:**
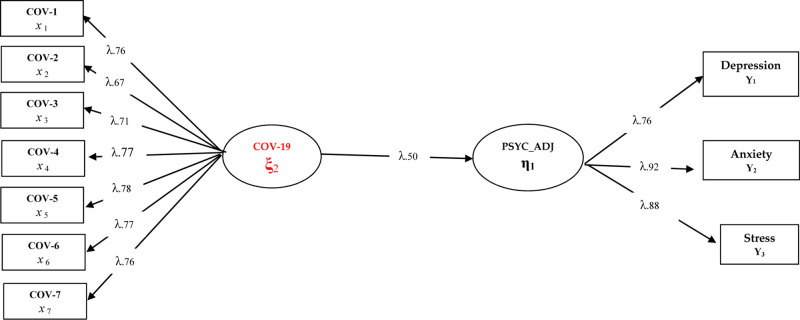
Standardized SEM results for Model 1.

Three different models were tested for the purposes of the research. In this context, the research hypothesis first constructed as Model 1 as “Fear of COVID-19 directly predicts psychological adjustment skills in healthcare professionals” was tested. In this model, fear of COVID-19 is expected to negatively and directly predict psychological adjustment skills in healthcare professionals. The findings regarding Model 1 are presented in Figure 1.

Considering the fit index values [χ^2^(44,26/34) = 1.30; CFI = 0.97; TLI = 0.96; NFI = 0.94;GFI = 0.93] of the model tested in Figure 1, it can be said that all the implicit variables in Model 1 have a significant relationship with the observed variables (*p* < 0.001). In addition, it is understood that the fear of COVID-19 has a negative predictive effect on psychological adjustment skills (β = 0.50, *p* < 0.01, 25%). This finding can be interpreted that the fear of COVID-19 has a strong and negative effect on health professionals’ psychological adjustment skills. In order to better understand the predictive coefficients between variables in structural equation models, it is recommended to examine the mediation relationships by including other possible variables. In this context, it is useful to examine the findings related to Model 2 and Model 3. Prior to the examination of other models, depending on the verification of the hypothesis tested in Model 1, the variables of experiential avoidance and psychological resilience were included in the related model. In this model, the effect of fear of COVID-19 on psychological adjustment skills was tested both directly and indirectly. In this context, Model 2 can be expressed as: How has the direct effect of COVID-19 fear on psychological adjustment skills in healthcare professionals changed after including experiential avoidance and psychological resilience in the model?

**FIGURE 2:**
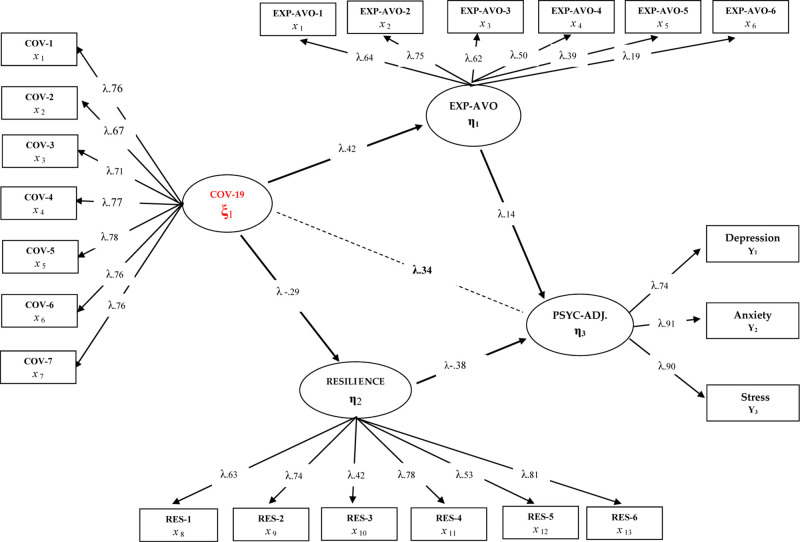
Standardized SEM results for Model 2.

**FIGURE 3:**
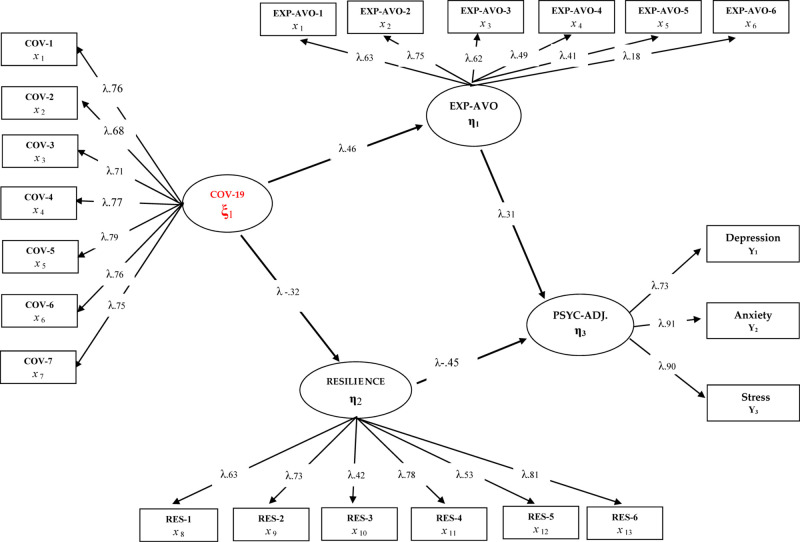
Standardized SEM results for Model 3.

Figure 2 shows the findings related to the structural model constructed as Model 2. In this sense, when the related model findings are analyzed, a significant change is observed in the direct correlation coefficients between the fear of COVID-9 and psychological adjustment skills with the inclusion of experiential avoidance and psychological resilience in the model. The general rule in the mediating relationships is that when the “mediating variable” is included in the model, there is a significant decrease in the direct predictive coefficients obtained in Model 1 (Tabachnick and Fidell, 2013). Accordingly, when Figure 2 is examined, it is seen that the direct predictive coefficient of the fear of COVID-19 on psychological adjustment skills is (β = 0.34, *p* < 0.01, 12%). However, the same predictive coefficients were determined in Model 1 as (β = 0.50, *p* < 0.01, 25%). These findings reached in Model 2 reinforce the idea that the variables included in the model may have an intermediary role. In addition, when Figure 2 is examined, it is understood that experiential avoidance has a negative effect and psychological resilience has a positive effect on psychological adjustment skills [χ^2^(456,30/204) = 2.23; CFI = 0.95; TLI = 0.95; SRMR = 0.060; RMSEA = 0.053]. Based on this finding, the direct predictive path from the fear of COVID-19 to psychological adjustment skills was removed from the model and thus the full mediation relationship was analyzed in order to test the full mediation relationship of these variables. Accordingly, Model 3 was constructed as follows; “Does the role of experiential avoidance and psychological resilience play a role in the relationship between fear of COVID-19 and psychological adjustment skills in healthcare professionals?”. The findings obtained are presented in Figure 3.

When Figure 3 is examined, it is seen that the tested model is well adapted and a significant change is obtained in the predictive coefficients of the variables whose mediation role is tested after removing the direct path from the fear of COVID-19 to psychological adjustment skills. In addition, when the fit indexes of the constructed model are examined, it can be said that they indicate a good level of fit [χ^2^(299.32/205) = 1.46; CFI = 0.98; TLI = 0.97; SRMR = 0.048; RMSEA = 0.046]. When the findings related to the mediation model are analyzed, the fear of COVID-19 has a positive relationship with experiential avoidance (β = 0.46, *p* < 0.01, 21%) and a negative relationship with psychological resilience (β = −0.32, *p* < 0.01, 10%). In the other dimension of the mediation model, it is seen that low psychological adjustment skills are positively predicted by experiential avoidance (β = 0.46, *p* < 0.01, 21%) and negatively by psychological resilience. There is also a significant increase in the mentioned predictive coefficients compared with Model 2. These findings can be interpreted that the impact of COVID-19 fear on low psychological adjustment skills in healthcare professionals was predicted indirectly by the variables of experiential avoidance and psychological resilience.

## Discussion

In this study, in which the effect of fear developed due to the COVID-19 pandemic in healthcare professionals on psychological adjustment skills was dealt in the context of experiential avoidance and psychological resilience, are discussed by considering the constructed models.

In this context, the first important finding reached within the context of the objectives of the research is the predictive role of the fear of COVID-19 on psychological adjustment skills in healthcare professionals. The fear developed in connection with COVID-19 has come to the forefront as an important pressure tool on depressive symptoms, anxiety and stress, which form psychological adjustment skills. During the pandemic with a traumatic nature, healthcare professionals are likely to be affected by the pandemic process and the adverse conditions they face in patients, both as an individual and as a professional (Greenberg et al., 2020; Schwartz and Graham, 2020). Banerjee (2020); Ornell et al. (2020), Shigemura et al. (2020), and Seçer and Ulaş (2020b) stated that the pandemic process should be considered as a traumatic difficult life process. In this regard, it can be thought that COVID-19 may affect psychological adjustment skills negatively in the short and long term by triggering intense stress, anxiety and depressive symptoms in healthcare professionals.

Although the negative effect of COVID-19 outbreak on psychological adjustment skills was determined during the research process, two different models were also tested by constructing the mediating roles of the variables of experiential avoidance and psychological resilience, which are thought to shape this effect significantly. When the mediating roles of these variables between the fear of COVID-19 and psychological adjustment skills were examined in the context of direct and indirect effects, it was seen that the predictive effect of the fear of COVID-19 on psychological adjustment skills occurred indirectly through these two variables. As a result of the mediation models, it was observed that the fear of COVID-19 put pressure on the experiential avoidance behavior in healthcare professionals, and experiential avoidance weakened their psychological adjustment skills. Hayes et al. (1996) defined experiential avoidance as reluctance to experience the negative feelings, thoughts, memories, and bodily feelings of the individual and avoidance reactions to reduce the frequency or effect of these experiences. Greenberg et al. (2020) stated that it is an important reflection of trauma. In this sense, it can be said that the intense fear associated with COVID-19 can direct the individual to dysfunctional avoidance responses, and this avoidance behavior will lead to various psychopathological symptoms (Ottenbreit and Dobson, 2004). Undoubtedly, the pandemic has created a psycho-socially challenging situation in healthcare professionals, just like everyone else, and this appears to be a significant risk factor in the psychological adjustment skills of healthcare professionals in the short and long term. It can be considered as an inevitable result that this effect causes emotional and behavioral problems in healthcare professionals either acutely or chronically (Orcutt et al., 2005; Briggs and Price, 2009; Hayes et al., 2012; Schwartz and Graham, 2020). This finding also shows consistency with the results of studies dealing with common disorders that are common in individuals with experiential avoidance. In this sense, emotional and behavioral problems such as low subjective well-being (Machell et al., 2015), eating disorders (Rawal et al., 2010), post-traumatic stress disorders (Orcutt et al., 2005), and depression (Briggs and Price, 2009) are common problems among those with a high level of experiential avoidance. In this regard, it can be thought that the negative psychological effect created by the COVID-19 outbreak will trigger traumatic experiential avoidance in healthcare professionals. As an important result of this, it is useful to take into account that healthcare professionals showing high levels of experiential avoidance can face various psycho-social adjustment problems.

It is thought that high levels of experiential avoidance may be associated with low psychological flexibility and this will put pressure on the individual’s adaptation skills (Bond et al., 2006). Psychological flexibility, which is put forward as one of the basic criteria of being healthy (Kashdan and Rottenberg, 2010), is defined as the flexibility and determination that an individual will show in order to cope with stressful and difficult life events, and achieve important life goals (Bond et al., 2006; Dalrymple and Herbert, 2007). Flexibility also guides the individual’s decisions and actions in this direction and strengthens the self-efficacy belief (Deci and Ryan, 2000). In this sense, it can be thought that those with high level of experiential avoidance will not be flexible enough and therefore will be deprived of effective coping and adaptation skills by displaying rigid behavioral patterns that lead to various psychopathologies (Kashdan and Rottenberg, 2010). In addition, clinical findings showed that having a low of level psychological flexibility, depression and social anxiety, etc. indicates that it significantly affects the healing process in disorders (Dalrymple and Herbert, 2007; Rüsch et al., 2008; Berking et al., 2009). Hence, it can be thought that low psychological flexibility (associated with experiential avoidance) may lead to a greater negative impact of the COVID-19 outbreak on healthcare workers. Therefore, examining the relationship between psychological flexibility and resilience can make important contributions to the literature.

Despite the short- and long-term risk of experiential avoidance on the psychological adjustment skills of healthcare professionals, it was determined that the level of psychological resilience of healthcare professionals has an important protective function. Psychological resilience is defined as the ability of the individual to recover in the face of difficult living conditions (Brooks et al., 2020a) and quickly return to his/her former and better status (Earvolino-Ramirez, 2007). In this respect, it has a psychological quality that healthcare professionals will need most during the epidemic process (Greenberg et al., 2020).

Findings obtained from the research reveal that fear of COVID-19 poses a risk for psychological resilience in healthcare professionals. In this sense, the high level of resilience appears to be a quality that protects the psychological adjustment skills of healthcare professionals while reducing the risk of COVID-19 on healthcare professionals. Therefore, it seems possible to limit or even prevent the negative impact of the fear and anxiety created by the epidemic on healthcare professionals through experiential avoidance-like features with the help of psychological resilience. In this sense, it is thought that emergency measures to improve the psychological resilience of healthcare professionals may contribute to the prevention of negative effects that may occur in the short and long term due to the epidemic. This will also strengthen the psychological adjustment skills of healthcare professionals and activate the effects that will strengthen their quality of life, life satisfaction and professional commitment.

### Limitations and Future Research

The findings of this research should be evaluated in the context of its limitations. The research was carried out only in a relational and cross-sectional context due to the negative effects caused by the pandemic. Data collection was also carried out online for the same reason and through convenient sampling method. The impact of these on research results should be taken into account. The research includes only on-the-job healthcare professionals who have not yet been infected. In this regard, it is thought that there is a need for studies involving healthcare professionals infected with the virus and recovered. In addition, it is considered that applying multimethod or mixed methods research in terms of data diversification will provide significant outcomes in the context of external validity. In addition, it is thought that studies focusing on comparisons between different countries may present important findings in terms of understanding the nature of the problem.

### Implications

The results of the research are considered to shed light on awareness of understanding the nature of the secondary effects that healthcare professionals will have depending on the epidemic and on prevention approaches to be used for the protection of healthcare professionals’ psychological health. In this case that the epidemic spread rapidly all over the world, it is considered that it will contribute to the understanding of the behavioral consequences of the emotional state developed due to COVID-19. Today, studies focusing on the secondary outcomes of the outbreak have gained momentum, and it is expected that similar research ideas will be created.

## Data Availability Statement

All datasets presented in this study are included in the article/supplementary material.

## Ethics Statement

The studies involving human participants were reviewed and approved by Gümüşhane University Health Sciences Ethics Committee. The patients/participants provided their written informed consent to participate in this study.

## Author Contributions

As a result of the review of the relevant literature, İS, ZK-Ö and SU acted together in the process of revealing the research idea. After determining the research subject, all authors took an active role in completing the research procedures. After obtaining the research permissions and ethics committee approvals, all authors conducted the data collection and analysis together. The process of creating online data collection processes and delivering them to the target groups was fulfilled together. The transfer of the data collected online to the SPSS environment and examination of its suitability for analysis and parametric test conditions were done by the İS and ZK-Ö. Data analysis and reporting processes were done by SU. In the writing process of the study, the introduction and discussion part was written by İS and ZK-Ö significantly and SU contributed to this process. The Methods and Findings section of the research were prepared for publication by ZK-Ö and contributed by İS and SU. The Discussion section is a section created by both authors together. During the publication of the manuscript, the feedback from the editors and the referees were organized together by all authors.

## Conflict of Interest

The authors declare that the research was conducted in the absence of any commercial or financial relationships that could be construed as a potential conflict of interest.

## Footnotes

1 https://forms.gle/DL7ojNSEbBGsAUNM6
